# Specificity and Actions of an Arylaspartate Inhibitor of Glutamate Transport at the Schaffer Collateral-CA1 Pyramidal Cell Synapse

**DOI:** 10.1371/journal.pone.0023765

**Published:** 2011-08-24

**Authors:** Weinan Sun, Katie M. Hoffman, David C. Holley, Michael P. Kavanaugh

**Affiliations:** Center for Structural and Functional Neuroscience, The University of Montana, Missoula, Montana, United States of America; Centre national de la recherche scientifique-, University of Bordeaux, France

## Abstract

In this study we characterized the pharmacological selectivity and physiological actions of a new arylaspartate glutamate transporter blocker, L-threo-ß-benzylaspartate (L-TBA). At concentrations up to 100 µM, L-TBA did not act as an AMPA receptor (AMPAR) or NMDA receptor (NMDAR) agonist or antagonist when applied to outside-out patches from mouse hippocampal CA1 pyramidal neurons. L-TBA had no effect on the amplitude of field excitatory postsynaptic potentials (fEPSPs) recorded at the Schaffer collateral-CA1 pyramidal cell synapse. Excitatory postsynaptic currents (EPSCs) in CA1 pyramidal neurons were unaffected by L-TBA in the presence of physiological extracellular Mg^2+^ concentrations, but in Mg^2+^-free solution, EPSCs were significantly prolonged as a consequence of increased NMDAR activity. Although L-TBA exhibited approximately four-fold selectivity for neuronal EAAT3 over glial EAAT1/EAAT2 transporter subtypes expressed in Xenopus oocytes, the L-TBA concentration-dependence of the EPSC charge transfer increase in the absence of Mg^2+^ was the same in hippocampal slices from EAAT3 +/+ and EAAT3 −/− mice, suggesting that TBA effects were primarily due to block of glial transporters. Consistent with this, L-TBA blocked synaptically evoked transporter currents in CA1 astrocytes with a potency in accord with its block of heterologously expressed glial transporters. Extracellular recording in the presence of physiological Mg^2+^ revealed that L-TBA prolonged fEPSPs in a frequency-dependent manner by selectively increasing the NMDAR-mediated component of the fEPSP during short bursts of activity. The data indicate that glial glutamate transporters play a dominant role in limiting extrasynaptic transmitter diffusion and binding to NMDARs. Furthermore, NMDAR signaling is primarily limited by voltage-dependent Mg^2+^ block during low-frequency activity, while the relative contribution of transport increases during short bursts of higher frequency signaling.

## Introduction

Five major subtypes of excitatory amino acid transporters exist in the CNS, and three of these (EAAT1-3; also known as GLAST, GLT-1, and EAAC1) are expressed in forebrain with distinct distribution patterns on astrocytes (EAAT1 and EAAT2) and neurons (EAAT3) [1]. Studies utilizing glutamate uptake inhibitors broadly indicate that the transporters play important roles in glutamate homeostasis, and that they can in some cases shape receptor dynamics during synaptic transmission [2]. While the synaptic effects of glutamate transport inhibition vary widely in different brain regions, studies of the hippocampal Schaffer collateral-CA1 pyramidal cell (SC-PC) synapse generally indicate that transporter activity does not strongly modify synaptic AMPAR responses [3,4; but see 5,6]. In contrast, extrasynaptic NMDAR activity is enhanced by glutamate uptake block in this region [7]–[11].

The relative contributions of the glial EAAT1 and EAAT2 and neuronal EAAT3 subtypes to restricting the spread of synaptically released glutamate from the SC-PC synapse is presently unclear. EAAT2 and EAAT3 are the dominant transporters in forebrain astrocytes and neurons, respectively, while EAAT1 is found on forebrain astrocytes at lower levels [12]. The widely used glutamate uptake blocker DL-TBOA blocks EAAT2 and EAAT3-mediated [^3^H]L-Glu uptake with IC_50_ values approximately seven-fold lower than for EAAT1 [13], and studies utilizing DL-TBOA indicate that it can induce spillover of synaptic glutamate onto NMDARs in hippocampus [7]–[11]. Selective inhibition of the postsynaptic neuronal transporter EAAT3 by intracellular ion substitution during whole cell recording or genetic manipulation also leads to augmentation of NMDAR-mediated EPSCs as well as changes in synaptic plasticity [9], [11]. Nevertheless there is still uncertainty surrounding the relative contributions and detailed roles of neuronal and glial EAAT subtypes in hippocampus. Another issue concerns the general role of glutamate transport in restricting NMDAR signaling in the hippocampus under physiological conditions, since this effect has only been reported in conditions permissive for channel activity, i.e. voltage clamp of the postsynaptic neuron at positive potentials or in Mg^2+^-free ACSF [7]–[11].

Threo-ß-benzylaspartate (TBA) is a new arylaspartate derivative that is structurally related to TBOA, but with a shorter aryl linkage lacking an ether group. In contrast to DL-TBOA, L-TBA displays moderate selectivity for the neuronal EAAT3 subtype over both EAAT1 and EAAT2 [14]. In this work we show that L-TBA does not cross-react with ionotropic glutamate receptors expressed on CA1 pyramidal cells at concentrations used to block uptake. We examined the effects of L-TBA on excitatory synaptic transmission at the Schaffer-CA1 synapse in wild-type and transgenic mice lacking the neuronal EAAT3 glutamate transporter in order to gain insights into the respective roles of glial and neuronal transporters. We also compared L-TBA actions on postsynaptic signaling with and without voltage clamp using whole-cell or extracellular recording in order to gain insights into the role of transporters in more physiologically relevant conditions. The data suggest that glial transporters restrict synaptically released transmitter binding to NMDARs to a significantly greater extent than neuronal transporters. Further, voltage-dependent Mg^2+^ block plays a dominant role in limiting NMDAR signaling during low-frequency activity, while the relative influence of transport increases during short bursts of activity.

## Methods

### Ethics statement

Mice and frogs used in this study were treated in a manner to minimize suffering, and were anesthetized with isofluorane or Tricaine respectively, and decapitated in accordance with NIH and University of Montana regulations. The study was approved by the University's IACUC (protocol approval 03905).

### Drugs

Drugs and chemicals were purchased from Sigma, except CNQX and DL-APV (Tocris) and DL-TBOA (Ascent). Stock solutions of TBOA and TBA were dissolved in DMSO at 50 mM. Data are presented as mean ± S.E. and statistical significance evaluated by Student's paired (drug effects) or unpaired (transgenic effect) t-test.

### Oocyte recording

Stage V Xenopus oocytes were microinjected with approximately 50 ng of human EAAT1, EAAT2, or EAAT3 cRNA and two-microelectrode voltage clamp recordings were made 3–5 days later at 22° with Molecular Devices amplifiers and A/D interfaces [14]. Oocytes were superfused with Ringer containing (in mM) 96 NaCl, 4 KCl, 1.8 CaCl_2_, 1.0 MgCl_2_, 5 HEPES pH 7.4 and were voltage clamped at −30 mV.

### Computational modeling

Human EAAT3 sequence (GenBank http://www.ncbi.nlm.nih.gov) was aligned with the Protein Data Bank (PDB) sequence of the archaeal homologue Glt_Ph_ [15], [16]. The EAAT3 homology model was constructed by threading the aligned sequence along PDB coordinates using the SwissProt server (http://swissmodel.expasy.org//SWISS-MODEL.html). The resulting model was optimized through local energy minimizations of regions with high steric and electrostatic interference using the AMBER7 force field in the Tripos SYBYL8.0 platform. Representations of L-TBA and L-TBOA were docked using GOLD v.3.0.1 (http://www.ccdc.cam.ac.uk/) into the EAAT3 model and evaluated using the ChemScore scoring function. Structures were seeded within a sphere of radius 8Å from the α-carbon of L-3-Br-TBOA in 2NWW. The structures from three separate docking run (30 seeds/run) were evaluated for electrostatic interactions with residues that have been shown to confer substrate and inhibitor specificity [15]–[18]. We screened docked structures for two interactions between EAAT3 R447 and inhibitor distal oxygens and between EAAT3 D444 and the inhibitor α-amino group. Structures with the lowest estimated ΔG values were incorporated into the homology model and represented using PyMol1.3.

### Mouse hippocampal slice preparation and recording

P18-26 CD1 wild-type or EAAT3 (−/−) siblings (Raymond Swanson, UCSF) were anesthetized with isofluorane and decapitated in accordance with University of Montana IACUC regulations (protocol approval number 039 05). The brain was rapidly dissected and placed in ice-cold solution containing (in mM): 80 NaCl, 24 NaHCO_3_, 25 glucose, 75 sucrose, 2.5 KCl, 1.25 NaH_2_PO_4_, 0.5 CaCl_2_, 5 MgCl_2_, 1 ascorbic acid, 3 Na pyruvate. The solution was saturated with 95% O_2_ and 5% CO_2_ (pH 7.3). 300 µm thick coronal hippocampal slices were cut using a vibratome (VT1200S Leica, Germany), then hemisected and placed in artificial cerebral spinal fluid (ACSF) containing (in mM): 126 NaCl, 2.5 KCl, 1.2 MgCl_2_, 2.4 CaCl_2_, 1.2 NaH_2_PO_4_, 11.4 glucose, and 21.4 NaHCO_3_ saturated with 95%O_2_ and 5% CO_2_ (pH 7.3) and maintained at 30°C. Slices were allowed at least 1 hour to recover before being placed in a submersion-type recording chamber perfused at 1.6–2.0 ml/minute with ACSF at 30°C. Slices were visualized on an upright fixed-stage microscope (Olympus BX51WI) equipped with infrared-differential interference contrast optics. The recording pipettes (3–6MΩ resistance) were filled with internal solution containing (in mM): 110 Cs methanesulfonate, 38 CsCl, 10 HEPES, 10 Na-phosphocreatine, 0.1 EGTA, 4.0 Mg-ATP, 0.3 GTP, 5 QX-314, pH = 7.3. Series resistance (typically 15–20 MOhm), was monitored and recording terminated if a change >20% was observed. Holding potential was −60 to −70 mV. 100 µM picrotoxin was added to the ACSF for whole-cell recordings and an incision was made between CA3 and CA1.

Extracellular field excitatory post synaptic potentials (fEPSPs) were recorded using glass electrodes filled with ACSF. Recordings were made with analog-digital converters and amplifiers from Molecular Devices, and data were acquired at 20 kHz and filtered at 5–10 kHz. Acquisition and analysis software was Axograph (version 1.1.6). EPSCs and fEPSPs were induced with 100 µs current pulses between 0.1 mA– 0.4 mA administered through ACSF-filled stimulating pipettes placed in stratum radiatum. EPSC charge transfer changes and fEPSP prolongation were quantified by integrating the area from the peak of the response (normalized to control) to 100 ms after the peak. Voltage-time integrals of the fEPSPs elicited by a burst of 2 or 3 stimuli were calculated after subtraction of responses to 1 and 2 stimuli, respectively. Whole cell recordings of synaptically activated transport currents (STCs) in CA1 astrocytes in stratum radiatum were made with assistance of fluorescence visualization of astrocytes following 20 min. incubation in 2 µM SR-101 (Sigma) and 1 h washout. STC recordings were made in ACSF including (in µM) 100 picrotoxin, 50 DL-APV, 20 CNQX, 10 8-CPT. Astrocytes were clamped at −90 mV (resting potential -80±1.5 mV; 9.1±.8 MOhm input resistance, n = 4). Peak current amplitudes were determined following baseline subtraction of persistent current at 200 ms following stimulation. The identity of the STC in astrocytes that was blocked by L-TBA was further confirmed by DL-TBOA [19] sensitivity.

Outside-out patches were pulled from the soma of CA1 pyramidal neurons from CD1 mice (P9-22) identified under transmitted IR DIC optics. Patches were held at −60 mV with a pipette solution containing (in mM): 150 K-gluconate, 10 HEPES, 8 NaCl, 0.5 EGTA, 4 MgATP, and 0.3 NaGTP, pH 7.3. Patch recordings were made at room temperature. Drug solution was applied through a 200 µm diameter theta tube attached to a piezoelectric bimorph. Solution change kinetics were estimated following patch rupture by measuring junction currents during switches between iso- and hypo-osmotic solutions as shown above current records. NMDAR currents were recorded in nucleated patches with Mg^2+^-free ACSF containing 20 µM CNQX and 20 µM glycine. L-glutamate with or without L-TBA (each at 100 µM) were applied in alternating order for 100 ms or 400 ms to induce AMPAR or NMDAR currents respectively. Averages of 5–10 responses to repetitive application are shown with an interpulse interval of 3 sec for AMPAR experiments and 10 sec for NMDAR experiments.

## Results

### Computational docking of L-TBA with glutamate transporters

**Figure 1:**
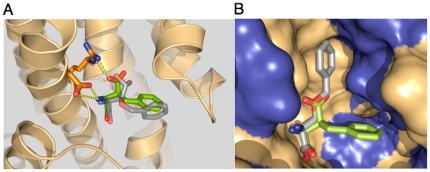
Interaction of L-TBA with EAATs. (A) Docking of L-TBA (green) and L-TBOA (gray) in EAAT3 model showing overlap of functional groups interacting with R447 and D444, with benzyl groups oriented toward extracellular loop HP2 as described in [14]. (B) Surface depiction of the transporter binding site (hydrophobic regions blue) showing L-TBA and alternate docking orientation of L-TBOA with benzyl ring aligned in alternate hydrophobic pocket.

We constructed an EAAT3 model using published homologous archaeal Glt_Ph_ structures [15], [16] and docked aryl-aspartate analogs in order to identify and compare plausible structural interactions. A single Na^+^ ion was positioned in the structure according to the structural determination of a Tl^+^ ion in the Glt_Ph_/L-3-Br-TBOA complex [15] and further corresponding to electrostatic predictions of Na^+^ ion binding sites in EAAT3 [20]. The EAAT3 residues R447 and D444 interact with the γ-carboxylate and the α-amino group of transported glutamate respectively, and these residues determine substrate specificity [17], [18]. The computationally docked L-TBA complex suggested corresponding electrostatic interactions between the blocker carboxyl and amino groups with the R447 and D444 residues in EAAT3 (Figure 1A). The most energetically favorable L-TBA complexes corresponded to the benzyl group orientation of L-3-Br-TBOA that was determined in the Glt_Ph_ crystal structure, with interactions between the ring and non-polar residues near the tip of HP2. Interestingly, when computational docking of L-TBOA was performed, an energetically favorable conformation was observed that involved an interaction of R447 with the ether group of the blocker. This alternate orientation positions the benzyl group in an ‘up’ conformation perpendicular to the membrane and parallel to TMD7, and aligns it with non-polar residues in TM7, TM8 and HP2 (Figure 1B). Docking energies (Table 1) predict that L-3-Br-TBOA could orient in either conformation while L-TBOA is predicted to predominantly align in the perpendicular, ‘up’ orientation, and L-TBA aligns predominantly in the ‘down’ conformation parallel to the membrane plane.

**Table 1:** Computational docking results.

Structure	ChemScore	ΔG (kJ mol^−^^1^)	orientation
L-TBOA	18.07	−22.14	perpendicular
L-TBOA	15.01	−18.62	parallel
L-TBA	−	−	perpendicular*
L-TBA	19.70	23.62	parallel
L-3-Br-TBOA	20.19	23.09	perpendicular
L-3-Br-TBOA	18.04	23.90	parallel

*structure not found by energy minimization algorithm.

### Interaction of L-TBA with neuronal and glial glutamate transporters

**Figure 2:**
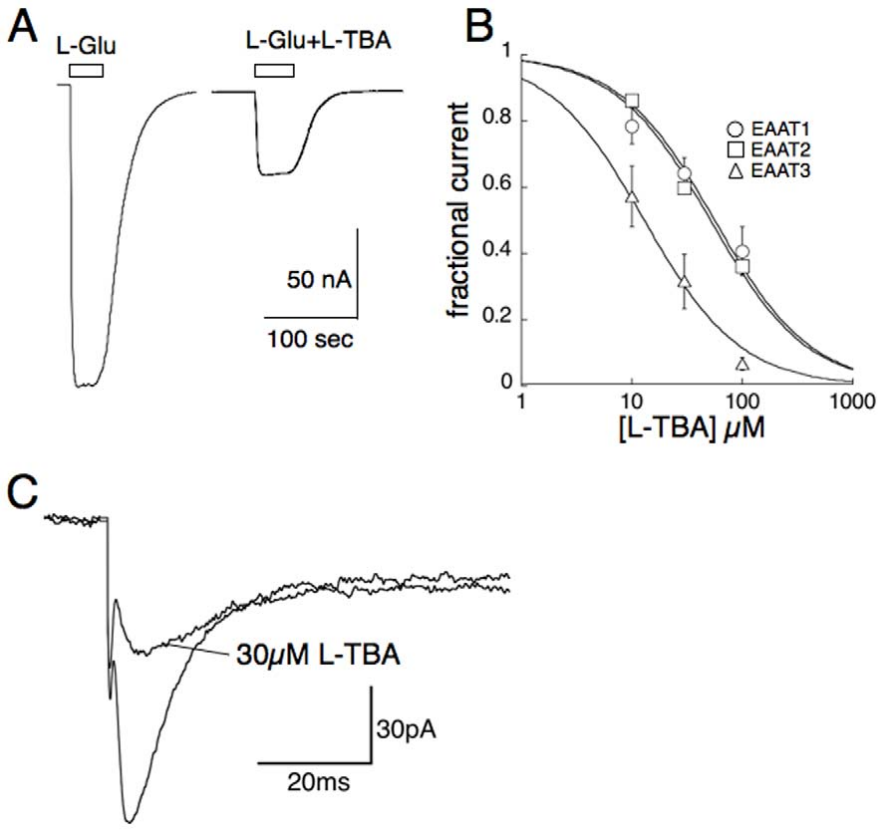
Effects of L-TBA on native and recombinant transporters. (A) Representative recording from voltage-clamped Xenopus oocyte expressing astrocyte transporter subtype EAAT2. 100 µM L-TBA partially blocks equimolar L-Glu uptake current mediated by EAAT2. (B) Summary of L-TBA concentration-dependence of block of 100 µM L-Glu currents in oocytes expressing EAAT1-3, showing approximately four-fold selectivity for EAAT3 by least-squares minimized fits to mean data generating IC_50_ values of 56, 52, and 13 µM, respectively. (C) Effect of 30 µM L-TBA on synaptically activated transport current (STC) in hippocampal CA1 astrocyte. Currents in the presence or absence of L-TBA were evoked by stimulation in stratum radiatum in the continuous presence of ionotropic receptor antagonists (see methods). 30 µM L-TBA blocked 66.7±10.4 of the peak STC (n = 4).

L-TBA inhibits uptake mediated by heterologously expressed EAATs with preference for the neuronal glutamate transporter subtype EAAT3 [14]. However, transport currents mediated by the major glial subtypes are also blocked in Xenopus oocytes expressing the transporters (Figure 2A,B). To examine the actions of L-TBA on glial transporters in situ, synaptically activated transport currents (STCs) were recorded in astrocytes in stratum radiatum of area CA1 in mouse hippocampal slices. Responses evoked by Shaffer collateral stimulation in the presence of the ionotropic receptor blockers CNQX (20 µM) and DL-APV (50 µM) revealed a current with properties consistent with glial transporters EAAT1 and EAAT2, together with a slowly decaying potassium current as previously described [21]. The STC, or transporter-mediated component of the evoked current, peaked and decayed within approximately 20 ms. The peak STC was blocked 67±10% by 30 µM L-TBA (n = 4; Figure 2C).

### Effects of TBA on fast excitatory synaptic transmission

**Figure 3:**
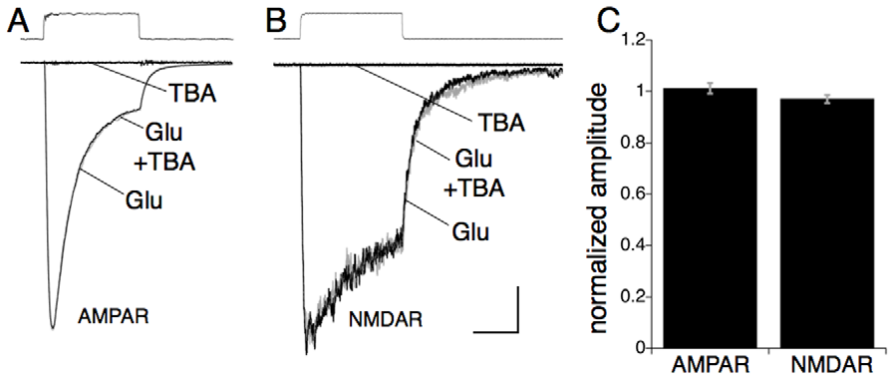
Representative recordings from outside-out patches excised from CA1 pyramidal neurons illustrating AMPAR and NMDAR responses to rapid application of 100 µM L-glutamate and/or 100 µM L-TBA for durations indicated by solution exchange traces above. Responses at −60 mV showing lack of agonist or antagonist actions of L-TBA on AMPARs (A; with 1.2 mM Mg^2+^) and NMDARs (B; with 0 mM Mg^2+^/20 µM glycine/20 µM CNQX). Scale bars are 50/200 ms and 50/100 pA for AMPAR/NMDAR responses respectively. (C) Summary of mean effects of 100 µM L-TBA on 100 µM L-Glu AMPAR and NMDAR responses.

Prior to characterizing the effects of L-TBA on excitatory synaptic transmission, its potential direct actions on ionotropic glutamate receptors were examined using a fast piezo solution switch to apply L- Glu and/or L-TBA to outside-out patches from CA1 pyramidal neurons. Fast application of 100 µM L-Glu induced robust AMPAR and NMDAR currents, while application of 100 µM L-TBA alone failed to induce measurable currents (Figure 3). Co-application of 100 µM L-TBA showed no antagonism of the AMPAR (101.2±2.0% of control; n = 4; p>0.6) or NMDAR currents (98.7±2.3% of control; n = 5, p = 0.59; Fig. 3C) induced by 100 µM L-Glu.

**Figure 4:**
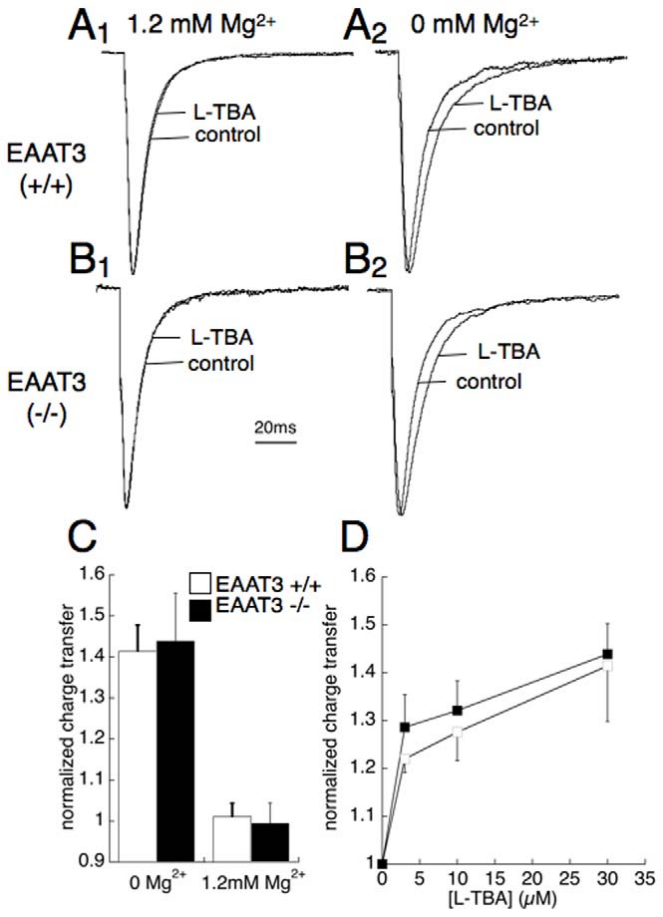
Actions of L-TBA (30 µM) on postsynaptic responses at the CA1 Schaffer collateral-pyramidal neuron synapse of EAAT3 +/+ (A) and EAAT3 −/− (B) mice. Representative whole cell recordings (−60 mV) showing effect of L-TBA on EPSCs evoked by stimulation in stratum radiatum in the presence (A_1_, B_1_) and absence (A_2_, B_2_) of physiological extracellular Mg^2+^ (1.2 mM). (C) Summary of data showing EPSC charge transfer increase in slices from EAAT3 (+/+) and (**−**/**−**) mice by 30 µM L-TBA in the absence and presence of Mg^2+^ (n = 5–7 slices; p<0.05). Data normalized to the charge transfer in slices from EAAT3 (+/+) mice. (D) Summary data showing no significant difference in L-TBA concentration-dependence of EPSC charge transfer increase (normalized to control) for EAAT3 +/+ (open squares) and EAAT3 **−**/**−** (filled squares) (n = 4).

The amplitude of fEPSPs elicited by .05 Hz stimulation in stratum radiatum was not affected by application of 30 µM L-TBA (100±3% of control; n = 41 slices). There was also no significant change in paired-pulse facilitation (50 ms pulse interval) of the peak amplitude of fEPSPs by application of L-TBA (control, 1.72±.03; L-TBA, 1.60±.08; p>.05). In whole cell voltage clamp recordings from CA1 pyramidal neurons, the effect of L-TBA on EPSC kinetics was highly [Mg^2+^]-dependent (Figure 4). In Mg^2+^-free ACSF, 30 µM L-TBA significantly prolonged evoked EPSCs. The charge transfer in the presence of L-TBA was 141±6% of that without drug (n = 6; p<.05), with no significant effect on the peak amplitude (102±7% of control, n = 6). The prolonging effect of L-TBA on EPSC kinetics was presumed to be mediated by NMDAR activity, because in the presence of physiological (1.2 mM) Mg^2+^, 30 µM TBA had no effect on the time course of the EPSC (charge transfer 101±3%; p = .78, n = 4; Fig.4A_1_). The prolongation was also not seen in the presence of 50 µM DL-APV in the absence of Mg^2+^ (data not shown). The effects of L-TBA on the EPSC charge transfer were concentration-dependent and not statistically different for slices from EAAT3 (+/+) and EAAT3 (-/-) mice (Figure 4C,D).

**Figure 5:**
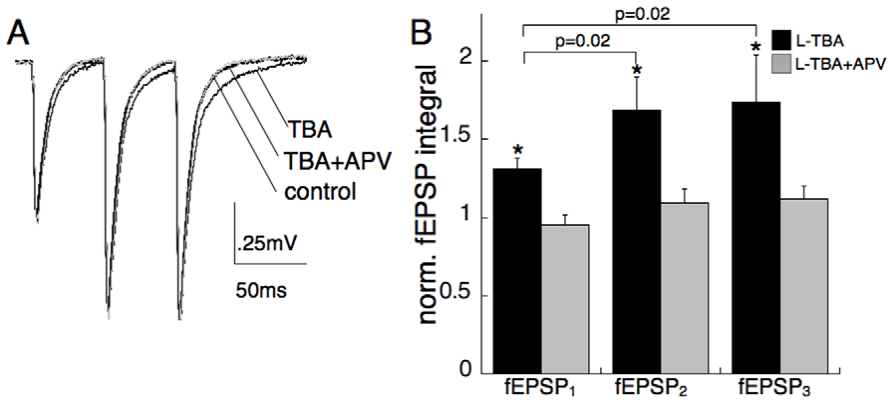
Actions of L-TBA on field responses at the CA1 Schaffer collateral-pyramidal neuron synapse. (A) Representative field EPSPs elicited in response to three stimuli delivered at 20 Hz in stratum radiatum. 30 µM L-TBA (black trace) prolonged fEPSPs relative to control (gray trace) in an activity-dependent manner (p = 0.02). The TBA prolongation was inhibited by co-application of 50 µM DL-APV (2nd black trace). (B) Summary of effects on fEPSP time-integrals elicited by 1, 2 and 3 stimuli normalized to corresponding fEPSPs in control ACSF (*p<.05 paired t-test; n = 9 slices for one and two stimuli, n = 5 slices for three stimuli).

Extracellular recording in the presence of physiological (1.2 mM) Mg^2+^ revealed an effect of L-TBA on fEPSP kinetics that exhibited pronounced frequency-dependence. 30 µM L-TBA slightly prolonged fEPSPs elicited by low-frequency (.05 Hz) stimulation, while time-integrals of fEPSPs recorded during a brief 20 Hz burst prolonged the time course of the fEPSP significantly further (Figure 5; 31±7% vs 69±21% for the first and second fEPSPs, respectively, n = 9, p = .02). Increasing stimulus strength to increase the fEPSP magnitude by an amount comparable to the frequency facilitation had no effect on the time course of the fEPSP (data not shown). The L-TBA-induced prolongation at both low and high frequencies was not observed in the presence of 50 µM APV, indicating that an increase in NMDAR signaling caused the fEPSP prolongation (Figure 5).

## Discussion

Selective glutamate uptake blockers are critical tools for studying the roles of glutamate transporters in modulating synaptic activity [6]–[11]. Both the widely used DL-TBOA (19) and the newer analog characterized in this study, L-TBA, are ß-substituted aryl aspartate analogs. The data demonstrate that L-TBA is selective for glutamate transporters over ionotropic glutamate receptors expressed on pyramidal neurons, as it neither antagonized ionotropic receptor responses to equimolar glutamate nor activated responses at concentrations up to 100 µM. Unlike TBOA, TBA lacks an ether linkage between the aryl group and the amino acid, resulting in a slight change in distance and bond angle of the benzyl ring relative to the aspartyl group (Figure 2A). The computationally predicted docking orientation of L-TBA was similar to the reported structure of L-3-Br-TBOA complexed with the archaeal homolog Glt_Ph_ [15], with key electrostatic interactions involving R447 and D444 in TMD7 of EAAT3. Predicted interactions of the benzyl group with hydrophobic regions of EAAT3 were also in agreement with the structure of L-3-Br-TBOA complexed with Glt_Ph_. In this conformation, the blocker prevents closure of the extracellular-facing HP2 loop which normally occludes bound L-aspartate (16). Interestingly, a distinct orientation was predicted for L-TBOA because of the alternate interaction of R447 with the ether oxygen of L-TBOA. This interaction preserves the α-amino group interaction with D444 but causes the benzyl group to rotate to an orientation perpendicular to the membrane plane, fitting into a hydrophobic domain bordered by TMDs 7/8 and HP2 (Figure 2B). These predicted conformations suggest that the HP2 loop position in the L-TBA and L-TBOA transporter complexes may slightly differ. In terms of transporter selectivity, L-TBOA exhibits moderate selectivity for the glial EAAT2 and neuronal EAAT3 subtypes, while L-TBA exhibits moderate selectivity for EAAT3 [13], [14]. While L-TBA and DL-TBOA differ in subtype selectivity, each exhibits significant subtype cross-reactivity at concentrations typically used. Because the effects of L-TBA on EPSC and fEPSP kinetics observed in this study were not significantly different in wild-type and transgenic mice lacking the EAAT3 gene, we conclude that they were primarily caused by inhibition of glial transporters EAAT1 and/or EAAT2, which would be predicted to be approximately 75% occupied at the inhibitor concentration used (30 µM) based on the K_D_ values of 12 µM and 9 µM for EAAT1 and EAAT2, respectively. This is roughly consistent with the 67±10% observed block of the peak synaptic transporter currents in astrocytes 30 µM L-TBA. While inhibition of glial transport appears to have predominantly mediated the effects on NMDARs we observed, it is important to note that selective block of neuronal transport has been reported to result in changes in both synaptic transmission and plasticity that were not addressed here [9], [11]. The mean increase in EPSC charge transfer caused by removal of extracellular Mg^2+^ was slightly larger in slices from EAAT3 (−/−) compared to wild-type mice, but this effect did not reach statistical significance (Figure 4C). The L-TBA concentration-dependance of the effects on NMDAR responses was also not statistically different in slices from EAAT3 (+/+) and EAAT3 (−/−) mice (Figure 4D). The threshold for the L-TBA response in both genotypes was ≤ 3 µM, suggesting that a relatively small fractional block of glial transporters (≤ 25%) affects excitatory postsynaptic responses to a greater extent than essentially complete block of neuronal transport.

Past work suggesting that transporters control extrasynaptic glutamate spillover has relied on examining the effects of transporter block in conditions where NMDAR activity is tonically enabled (i.e. under voltage clamp at depolarized potentials or with Mg^2+^-free solution). Consistent with work from several groups [6]–[11], we found that EAAT inhibition significantly prolonged EPSCs recorded at the Schaffer-CA1 pyramidal cell synapse in Mg^2+^-free conditions due to enhanced NMDAR signaling. L-TBA had no effect on postsynaptic responses in voltage clamp conditions with NMDARs blocked by physiological [Mg^2+^]. Because voltage-dependent Mg^2+^ block of NMDARs is dynamic during synaptic transmission, gaining greater insight into the role of glutamate transport in modulating synaptic activity will require the use of selective transport blockers in physiological conditions without voltage clamp. In this study, we have begun to address this issue and have shown that NMDAR-mediated components of fEPSPs can be isolated that are dependent on glutamate transporter activity in a frequency-dependent manner. The small effect of L-TBA on the kinetics of fEPSPs elicited by low-frequency stimulation in physiological Mg^2+^ was increased during brief bursts of higher frequency synaptic activity, and this prolongation was blocked by the NMDAR antagonist APV. This effect was not likely to be due simply to frequency-facilitation of transmitter release, because increasing stimulus strength did not affect the kinetics of fEPSPs elicited by low frequency stimulation. This suggests that the influence of glial glutamate transporters on NMDAR signaling may vary with synaptic frequency through postsynaptic voltage responses. A deeper quantitative understanding of the role of glutamate transporters in excitatory synaptic transmission will require further studies accounting for these variables.
